# Severe Pulmonary Hypertension in a Patient with Scurvy: Can a Vitamin Reverse It?

**DOI:** 10.1155/2021/5519937

**Published:** 2021-03-30

**Authors:** Vandan Shah, Ruchit N. Shah, Lauren Greene, Lauren M. DiMarino

**Affiliations:** ^1^Geisinger Medical Center, 100 N Academy Ave, Danville, PA 17822, USA; ^2^Department of Gastroenterology, 100 N Academy Ave, Danville, PA 17822, USA

## Abstract

**Introduction:**

Pulmonary hypertension secondary to scurvy is a rare manifestation that historically has not been well studied and is only described in a handful of case reports.

**Case:**

Our case is about a 35-year-old female with a history significant for drug and tobacco abuse, obesity, poor diet, anxiety, and major depressive disorder who was found to have severe pulmonary hypertension in the setting of vitamin C deficiency (<0.01 mg/L).

**Conclusion:**

We present a case that shows pulmonary hypertension can be associated with scurvy and reversed rapidly with adequate vitamin C supplementation.

## 1. Introduction

In the United States and other developed countries, it is often assumed the incidence of vitamin C deficiency or scurvy is negligible due to widespread accessibility to fortified foods and vitamins. However, individuals who live alone and have a history of substance abuse, restrictive diet, or mental illness are much more likely to develop scurvy [1]. Common early manifestations of scurvy include anemia, bone pain, easy bruisability, petechiae, gum disease, perifollicular hemorrhages on lower extremities, and mood alterations [2]. A rare manifestation is pulmonary hypertension. A literature review from January 2020 found 10 cases written about pulmonary hypertension due to vitamin C deficiency. In all these cases, there was rapid resolution of the patient's pulmonary hypertension after supplementation with vitamin C [3]. However, there are not any current guidelines or long-term follow-ups (maximum 1 year) to treatments documented. It is thought that vitamin C deficiency leads to pulmonary hypertension secondary to decreased degradation of hypoxia-inducible factor (HIF) and decreased activity of nitrous oxide synthase (NOS) [4, 5]. Our case is about a 35-year-old female who was found to have severe pulmonary hypertension in the setting of scurvy.

## 2. Case Report

**Figure 1:**
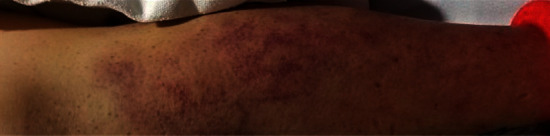
Peripheral ecchymosis which is commonly found as a cutaneous manifestation of scurvy.

**Figure 2:**
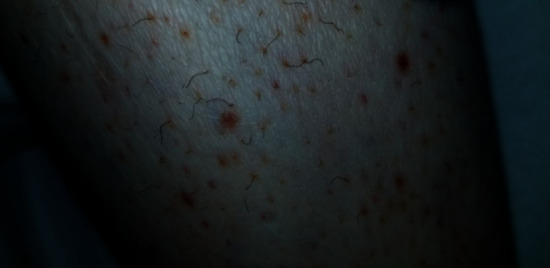
Corkscrew hair and perifollicular petechiae which are commonly seen in scurvy.

Our patient was a 35-year-old female with a past medical history significant for obesity, substance abuse, tobacco abuse (22-pack-year history), major depressive disorder, anxiety, and poor diet (primarily cheese steaks, chicken, and pork chops) who was admitted to our hospital with painful lower extremity swelling, shortness of breath, gingival bleeding, decreased oral intake, and ecchymosis which had been progressively getting worse over the past 6 months. The patient's course prior to admission included failed treatment with corticosteroids for suspected vasculitis/undifferentiated connective tissue disease and multiple transfusions in the setting of persistent anemia. The patient presented with a heart rate at 105 beats per minute, blood pressure of 115/59, respiratory rate at 24 breaths per minute, and oxygen saturation of 99% on room air. Physical exam revealed an extremely pale-appearing individual who had poor dental hygiene with mild pooling of blood in the oral cavity, diffuse joint swelling with ecchymosis (Figure 1), perifollicular petechiae with some corkscrew hairs (Figure 2), tenderness in the lower extremities, and decreased breath sounds with mild crackles.

The patient's labs were significant for hemoglobin: 6.6 g/dl (ref: 12.0–15.3 g/dl), prothrombin time/internalized normalized ratio (PT-INR): 1.50 (ref: 0.87–1.17), platelets: 152 K/uL (ref: 140–400 K/uL), brain natriuretic peptide (BNP): 2714 pg/ml (ref: 0–299 pg/ml), folate <2.0 ng/ml (ref: >4.5 ng/ml), and thiamine <7 nmol/L (ref: 8–30 nmol/L). Blood cultures came back positive for *Streptococcus anginosus* in 2/4 bottles, for which she was initially treated with amoxicillin/sulbactam and later transitioned to amoxicillin/clavulanic acid for a total of 14 days. In the setting of persistent anemia and acute worsening thrombocytopenia, a bone marrow biopsy was done and showed dyserythropoiesis, nuclear cytoplasmic asynchrony, and erythroid hyperplasia. A peripheral blood smear described increased anisocytosis of the red blood cells, left-shifted neutrophils with immature forms present, and small-to-medium platelets with normal morphology. A ventilation/perfusion scan was done which ruled out pulmonary embolism. Full vasculitis and viral workup was negative. Urine toxicology screen was positive for benzodiazepines and morphine/codeine.

**Figure 3:**
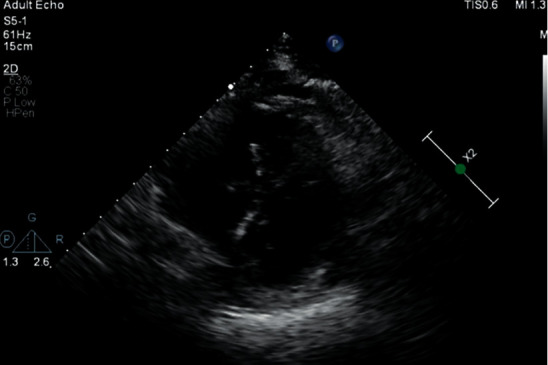
This image was obtained by the transthoracic echocardiogram using a parasternal short-axis view. There is increased pressure/volume in the right ventricle and flattening of the interventricular septum creating a D-shaped right ventricle (known as a D-sign). In our case, this right ventricular strain is secondary to increased pressure in the pulmonary vasculature.

Given her constellation of symptoms and poor nutrition, scurvy was on the differential and was started on daily vitamin C supplementation. A transthoracic echocardiogram (TTE) was ordered in the setting of bacteremia, which was negative to vegetation but was found to have severe pulmonary hypertension with a pulmonary artery systolic pressure of 86 mmHg, dilated inferior vena cava with a right atrial pressure of 15 mmHg, and severely enlarged right ventricle with flattening of the interventricular septum positive for D-sign (Figure 3). The left ventricle was concentrically hypertrophic with an ejection fraction of 70% and had a left ventricular outflow tract septal obstruction from an enlarged right ventricle. These findings further decreased suspicion for septic cardiomyopathy as TTE findings did not correlate and the patient continued to have symptoms despite antibiotic treatment. Although rare, thoracic medicine suspected the pulmonary hypertension was secondary to scurvy, and it would resolve with vitamin C supplementation as had been described in a few case reports. The decision was made to defer starting diuretic therapy or vasodilators. Ascorbic acid levels returned severely deficient two days after initiation of supplements at <0.1 mg/L, ref: 0.3–2.7 mg/dl. Within a week, the patient reported improvement in her shortness of breath, lower extremity tenderness, and gingival bleeding. She was discharged with vitamin C 500 mg twice daily and a follow-up right heart catheterization with TTE two weeks after initial initiation of supplementation.

Repeat TTE was negative for any indications of pulmonary hypertension: the right ventricle size and right atrial pressure (3 mmHg) were normal. Right heart catheterization had a baseline mean pulmonary artery pressure (mPAP) of 19, wedge pressure of 10, and pulmonary vasculature resistance (PVR) of 1 WU. Postsaline mPAP was 29, wedge pressure was 15, and PVR was 1.7 WU. Findings did not meet criteria for pulmonary hypertension, however did suggest mild noncompliance of the pulmonary vasculature that can be explained by mild left ventricular diastolic dysfunction.

The patient was assessed by cardiology two years later with resolution of her shortness of breath and no evidence of pulmonary hypertension noted on examination.

## 3. Discussion

Pulmonary hypertension is a rare symptom of vitamin C deficiency (scurvy) that has not been thoroughly investigated with documentation only in a handful of case reports. In our case, the patient's severe pulmonary hypertension resolved after 2 weeks of vitamin C supplementation. When comparing our patient's presentation with previous cases, there were similarities such as dilation of the right ventricle, elevated BNP, and rapid resolution of pulmonary hypertension with vitamin C supplementation [3]. Unlike other cases documented in the past, our case can confirm continued resolution of pulmonary hypertension at least two years after initiation of supplementation.

The exact mechanism of how scurvy leads to pulmonary hypertension is still unclear. One theory is that vitamin C acts as a reducing agent to activate EGLN prolyl hydroxylase which in turn hydroxylates HIFa. Hydroxylated HIFa is then targeted by von Hippel–Lindau protein for ubiquitination and proteasomal degradation. When vitamin C is low, there are increased levels of HIFa which binds with transcription factor HIFb to upregulate transcription of vasoconstrictive genes such as vasoconstrictor endothelin (EDN1) that increase pulmonary vasoconstriction and smooth muscle proliferation [4, 6]. This mechanism is similar to vitamin C's role as a cofactor for prolyl and lysyl hydroxylases for collagen synthesis, where a lack of it is attributable to many of scurvy's common manifestations such as poor wound healing, gingival bleeding, and arthralgia [7]. Vitamin C has also been shown to increase nitrous oxide synthase (NOS) activity by degrading asymmetric dimethyl L-arginine (NOS inhibitor), inhibiting arginase (increased L-arginine to be used to make NO), and increasing the availability of tetrahydrobiopterin (BH_4_) (essential cofactor of NOS). Therefore, vitamin C deficiency leads to decreased availability of NO and consequently pulmonary vasoconstriction and increased vascular tone [5, 8, 9].

In developed countries, smoking history, long-term alcoholism, low income, obesity, psychiatric illness, old age, and voluntarily restrictive diets are risk factors for scurvy [1, 10]. In particular, smokers have increased risk of attaining pulmonary hypertension secondary to scurvy as they have three times less vitamin C than nonsmokers and have higher incidences of hypoxia which also increase the levels of HIF [4, 11]. Our patient had a long-term history of smoking, psychiatric illness, poor diet, and poor oral intake which all contributed to her vitamin C deficiency. The presumed rarity of the disease may also be inaccurate as some studies have shown the prevalence of vitamin C deficiency can be as high as 25% in low-income families [12]. It is therefore important to keep in mind these risk factors so that diagnosis and treatment can occur early before irreversible damage occurs.

## 4. Conclusion

In conclusion, we present a case of pulmonary hypertension secondary to vitamin C deficiency that rapidly resolved and continued to be resolved two years later with supplementation. As there are no current guidelines recommending looking for vitamin C deficiency as a culprit for pulmonary hypertension, it is likely that many cases are missed. We believe that all individuals with the risk factors outlined above who are found to have pulmonary hypertension should be screened for vitamin C deficiency.

## Data Availability

The data used to support the findings of this study are included within the article.
